# Risk factors for central lymph node metastasis in patients with papillary thyroid carcinoma: a retrospective study

**DOI:** 10.3389/fendo.2023.1288527

**Published:** 2023-11-17

**Authors:** Jiachen Du, Qing Yang, Yixuan Sun, Peng Shi, Hao Xu, Xiao Chen, Tianyi Dong, Wenjing Shi, Yatong Wang, Zhenzhi Song, Xingchen Shang, Xingsong Tian

**Affiliations:** ^1^ Department of Breast and Thyroid Surgery, Shandong Provincial Hospital, Shandong First Medical University, Jinan, Shandong, China; ^2^ Department of Hematology, Shandong Provincial Hospital, Shandong First Medical University, Jinan, Shandong, China; ^3^ Department of Breast and Thyroid Surgery, Shandong Provincial Hospital, Shandong University, Jinan, Shandong, China

**Keywords:** papillary thyroid carcinoma, central lymph node metastasis, *BRAF* V600E mutation, prognosis, nomogram

## Abstract

**Introduction:**

Thyroid cancer is the most prevalent endocrine malignancy, with its global incidence increasing annually in recent years. Papillary carcinoma is the most common subtype, frequently accompanied by cervical lymph node metastasis early on. Central lymph node metastasis (CLNM) is particularly the common metastasis form in this subtype, and the presence of lymph node metastasis correlates strongly with tumor recurrence. However, effective preoperative assessment methods for CLNM in patients with papillary thyroid carcinoma (PTC) remain lacking.

**Methods:**

Data from 400 patients diagnosed with PTC between January 1, 2018, and January 1, 2022, at the Shandong Provincial Hospital were retrospectively analyzed. This data included clinicopathological information of the patients, such as thyroid function, BRAF V600E mutation, whether complicated with Hashimoto’s thyroiditis, and the presence of capsular invasion. Univariate and multivariate logistic regression analyses were performed to assess the risk factors associated with cervical CLNM in patients with PTC. Subsequently, a clinical prediction model was constructed, and prognostic risk factors were identified based on univariate and multivariate Cox regression analyses.

**Results:**

Univariate and multivariate analyses identified that age >45 years (P=0.014), body mass index ≥25 (P=0.008), tumor size ≥1 cm (P=0.001), capsular invasion (P=0.001), and the presence of BRAF V600E mutation (P<0.001) were significantly associated with an increased risk of CLNM. Integrating these factors into the nomogram revealed an area-under-the-curve of 0.791 (95% confidence interval 0.735–0.846) and 0.765 (95% confidence interval: 0.677–0.852) for the training and validation sets, respectively, indicating strong discriminative abilities. Subgroup analysis further confirmed that patients with papillary thyroid microcarcinoma and BRAF V600E mutations who underwent therapeutic central compartment neck dissection had significantly better 3-year disease-free survival than those who had prophylactic central compartment neck dissection (P<0.001).

**Conclusion:**

The study revealed that age >45 years, body mass index ≥25, tumor size ≥1 cm, BRAF V600E mutation, and capsular invasion are the related risk factors for CLNM in patients with PTC. For patients with clinically nodal-negative (cN0) papillary thyroid microcarcinoma, accurately identifying the BRAF V600E mutation is essential for guiding the central lymph node dissection approach and subsequent treatments.

## 1 Introduction

Thyroid cancer is a common endocrine malignancy, accounting for 3–4% of all malignant tumors. In recent years, the widespread adoption of neck ultrasonography and fine-needle aspiration (FNA) has increased the detection of microcarcinomas, elevating its incidence over the past decade (1). Based on pathological types, thyroid cancer can be classified into papillary, follicular, medullary, and anaplastic carcinomas, with papillary carcinoma accounting for 80–90% of cases (2). Lymph node metastasis, specifically into central and lateral nodes, is the primary route of metastasis in papillary carcinoma. The central lymph nodes include the prelaryngeal (Delphian), pretracheal, paratracheal, and tracheoesophageal groove lymph nodes, whereas the lateral nodes include cervical II, III, IV, and V level lymph nodes. In addition to conventional factors, such as age, sex, and body mass index (BMI), there is increasing consensus by experts and scholars on the influence of Hashimoto’s thyroiditis (HT) and *BRAF* V600E mutations on central lymph node metastasis (CLNM) in patients with papillary thyroid carcinoma (PTC). However, many related factors remain controversial. The increasing incidence of thyroid cancer is closely related to the clinical diagnosis of indolent, low-risk micropapillary carcinomas. Papillary thyroid microcarcinoma (PTMC) is defined as a tumor with a diameter of ≤ 1 cm in PTC (3). Various studies since the 21st century have identified different risk factors for CLNM in PTC. Some studies suggested demographic factors, such as age, sex, and BMI, as potentially associated with CLNM, and others propose genetic factors, such as HT and *BRAF* V600E mutations (4–7). Nevertheless, many factors affecting CLNM and the underlying mechanisms in patients with PTC remain unclear. The lymph node status is a critical prognostic factor in PTC that prioritizes understanding the risk factors for CLNM (8). Notably, one out of every 20 women is diagnosed with this disease. Despite its prevalence, the mortality rate remains relatively low, 0.5 and 0.3 per 100,000 for women and men, respectively (9). PTC, originating from thyroid follicular cells, is the most common thyroid malignant tumor. The age of onset varied from 30 to 40 years, with a 10-year survival rate exceeding 95% (10). CLNM is the primary metastatic route for PTC and a significant risk factor for recurrence in patients with PTC (11). In current clinical diagnosis and treatment, the prevailing diagnostic approach involves subjecting suspicious malignant lesions and abnormally enlarged lymph nodes identified in ultrasound reports to FNA+*BRAF* V600E testing. The results are crucial in determining the surgical approach and evaluating prognosis. PTC is characterized as an indolent malignant tumor, and surgical interventions can resolve most conditions; however, certain cases may cause life-threatening recurrence (12). During surgical interventions, central lymph node dissection could affect local recurrence and long-term survival. Without or incomplete dissection might elevate the risk of recurrence, diminish the patient’s quality of life, or adversely impact prognosis.

This study aimed to identify significant risk factors affecting CLNM through comprehensive analysis. This will enable the refinement of a more thorough central lymph node dissection surgical approach for high-risk groups, minimizing recurrence and metastasis rates. In addition to age, sex, and BMI, we examined the relationship between HT and *BRAF* V600E mutations and CLNM in patients with PTC. Our study focuses on the occurrence of CLNM, the number of central lymph nodes dissected, and the number of pathologically confirmed metastatic central lymph nodes as we establish a predictive model based on these relevant risk factors.

## 2 Materials and methods

### 2.1 Study population and design

We collected clinical data from 400 patients who underwent FNA+*BRAF* V600E gene testing and partial or total thyroidectomy with central lymph node dissection between January 1, 2018, and January 1, 2022, at Shandong Provincial Hospital. We used the 8th version of the AJCC staging system during our research process. Written informed consent for FNA+*BRAF* V600E gene testing was obtained from all patients. All procedures described in this study comply with the ethical standards of the institution.

Inclusion criteria for the patients in this study were: (1) Confirmed diagnosis of PTC through postoperative diagnostic pathology with paraffin-embedded tissues; (2) Patients with PTC who underwent preoperative FNA+*BRAF* V600E gene testing; (3) Central lymph node dissection performed during surgery, with diagnostic pathology using paraffin-embedded tissues reporting lymph node metastasis; (4) Availability of complete demographic, disease, and genetic data; (5) Age ≥18 years; (6) Absence of other systemic malignant tumors.

Patients were excluded from the study if they had any of the following: (1) Postoperative diagnostic pathology with paraffin-embedded tissues confirmed non-PTC or mixed-type PTC (2) Absence of preoperative FNA+*BRAF* V600E gene testing; (3) Central lymph node dissection not performed during surgery or the absence of lymph node metastasis information in the diagnostic pathology report with paraffin-embedded tissues; (4) Loss to follow-up; (5) Previous neck radiotherapy or a history of familial tumors; (6) Reoperation; (7) Pathological or clinical diagnosis of distant metastasis. We included 400 patients in this study.

Similarly, we analyzed the age, sex, BMI, thyroid function test (free triiodothyronine 3, free triiodothyronine 4, and thyroid stimulating hormone), carcinoembryonic antigen, presence of *BRAF* V600E mutation, whether combined with HT, number of PTC lesions, tumor size (cm), presence of capsular invasion, and the intraoperative pathological results of the central lymph node dissection.

### 2.2 Identification of the *BRAF* mutation status

The *BRAF* gene was investigated using the fluorescence quantitative Amplification Refractory Mutation System Polymerase Chain Reaction, with V600E1/K/E2/R/D1/D2 as the detection sites. The tests were performed at the Department of Pathology, Shandong Provincial Hospital. All samples were FNA specimens obtained from the Ultrasound Department of Shandong Provincial Hospital. Samples were evaluated against a cutoff concentration, and the presence of mutations at any of the E1/K/E2/R/D1/D2 sites indicated *BRAF* gene mutation.

### 2.3 Treatments

The treatment of malignant tumors, ranging from surgery to radioactive iodine I131, was standardized for all patients following the consensus of the multidisciplinary oncology committee. The histological classification adhered to that of WHO. According to the *Guidelines for the diagnosis and management of differentiated thyroid cancer in Chin*a, all patients with thyroid cancer underwent routine central lymph node dissection. When thyroid nodules resembled malignancy on preoperative ultrasound and were subsequently confirmed as malignant via FNA+*BRAF* V600E testing, these patients were subjected to thyroid lobectomy with isthmusectomy/total thyroidectomy. Prophylactic central compartment neck dissection (pCCND) was conducted for patients diagnosed with clinically nodal-negative (cN0) PTC, whereas therapeutic central compartment neck dissection (tCCND) was conducted for those identified with clinically nodal-positive (cN1) PTC. The anatomical boundaries and composition of the central lymph nodes were defined as the upper boundary being the hyoid bone, the lower boundary set at the level of the innominate artery, the lateral boundary by the common carotid artery, the anterior boundary by the investing layer of the deep cervical fascia, and the posterior boundary by the prevertebral fascia. This region comprises the prelaryngeal (Delphian), pretracheal, and left and right tracheoesophageal groove lymph nodes.

### 2.4 Postoperative follow-up

All patients with PTC included in this study underwent postoperative follow-up from the date of surgery until June 2023. The study’s endpoint was postoperative recurrence from the date of tumor resection and central lymph node dissection to the second surgery (all recurrent patients received a secondary surgery). Recurrence was defined as an abnormal enlarged lymph node or an abnormal lesion in the initial thyroid location observed on a thyroid ultrasound during routine reexamination, with cancer cells identified through FNA, and a diagnostic pathology from paraffin-embedded tissues post-second surgery confirming it as PTC.

### 2.5 Statistical analysis

Continuous variables were compared using the Unpaired Student’s t test or Wilcoxon rank-sum test, and categorical variables were compared with the Chi-square test or Fisher’s exact test. All tests were two-sided, and a p-value <0.05 was considered significant. Disease-free survival (DFS) of patients was evaluated using the Kaplan–Meier method, and differences were assessed with the log-rank test. All data analysis and plotting were performed using R software version 4.0.3. Non-parametric tests were used for inter-group comparisons when testing for differences between independent and dependent variables. Variables that showed statistical differences in univariate tests were subsequently incorporated into the binary logistic regression model for further analysis.

## 3 Results

### 3.1 Baseline information of PTC patients

This study included 400 patients diagnosed with PTC through postoperative pathological examinations. Of these patients, 81.25% were female (325 females and 75 males). Moreover, 29% were aged >45 years (116 individuals) and 49.5% had a BMI ≥25 (198 individuals). Capsular invasion was observed in 5.25% of tumors (21 individuals), and tumors sized ≥1 cm accounted for 20.25% of the patients (81 individuals). The *BRAF* V600E gene mutation was present in 79% of patients (316 individuals), and CLNM was identified in 35% (140 individuals).In total, 2181 LNs were dissected, of which 381 LNs were metastases (17.4%). The rate of metastatic LNs was 35% (140/400) (**Table 1**).

**Table 1:** Baseline characteristics.

Characteristics	All patients (n = 400)
Sex	
Female	325(81.25%)
Male	75(18.75%)
Age	
≤45	284(71.00%)
>45	116(29.00%)
BMI	
<25	202(50.50%)
≥25	198(49.50%)
Hashimoto’s thyroiditis	
YES	117(29.25%)
NO	283(70.75%)
Focality	
Multifocal	106(26.50%)
Unifocal	294(73.50%)
Size	
<1cm	319(79.75%)
≥1cm	81(20.25%)
Central lymph node metastasis	
YES	140(35.00%)
NO	260(65.00%)
Capsular invasion	
YES	21(5.25%)
NO	379(94.75%)
BRAF	
(+)	316(79.00%)
(-)	84(21.00%)
FT3	4.19 ± 0.53
FT4	11.89 ± 4.13
TSH	2.33 ± 1.87
CEA	1.69 ± 3.17
Surgical Procedures	
pCCND (cN0)	190(47.50%)
tCCND (cN1)	210(52.50%)

BMI, Body Mass Index; FT3, free triiodothyronine; FT4, free thyroxin; TSH, thyroid stimulating hormone; CEA, carcinoembryonic antigen; pCCND, Preventive Central Lymph Node Dissection; tCCND, therapeutic Central lymph node dissection.

According to the surgical date, 400 patients were divided into a training group and a validation group in a 7:3 ratio, with the final 30% of patients being included in the validation group. The baseline characteristics of these patients are shown in S. Statistical comparisons were performed to ensure the absence of significant differences in the clinical and pathological baseline data between these cohorts.

### 3.2 Association of CLNM and clinic-pathological characteristics

We evaluated all patients with PTC by conducting a univariate analysis of the effects of various factors on CLNM and found significant associations of CLNM with age >45 years (P=0.014), BMI ≥25 (P=0.008), tumor size ≥1 cm (P=0.001), capsular invasion (P=0.001), and the presence of the *BRAF* V600E mutation (P<0.001). In contrast, no significant associations of CLNM were observed for the variables of whether combined with HT (P=0.606) or the number of lesions (P=0.469) (**Supplementary Table 1**).

### 3.3 Multivariate logistic regression analysis

In the multivariate analysis, we found that age >45 years (odds ratio [OR]=2.153, 95% confidence interval [CI]: 1.166–3.974, P=0.014), BMI ≥25 (OR=2.165, 95% CI: 1.224–3.829, P=0.008), size ≥1 cm (OR=3.123, 95% CI: 1.606–6.074, P=0.001), capsular invasion (OR=29.183, 95% CI: 4.152–205.138, P=0.001), and *BRAF* V600E mutation (OR=7.924, 95% CI: 2.683–23.402, P<0.001) were significantly associated with CLNM (**Supplementary Table 3**).

### 3.4 Modeling and validation

**Figure 1:**
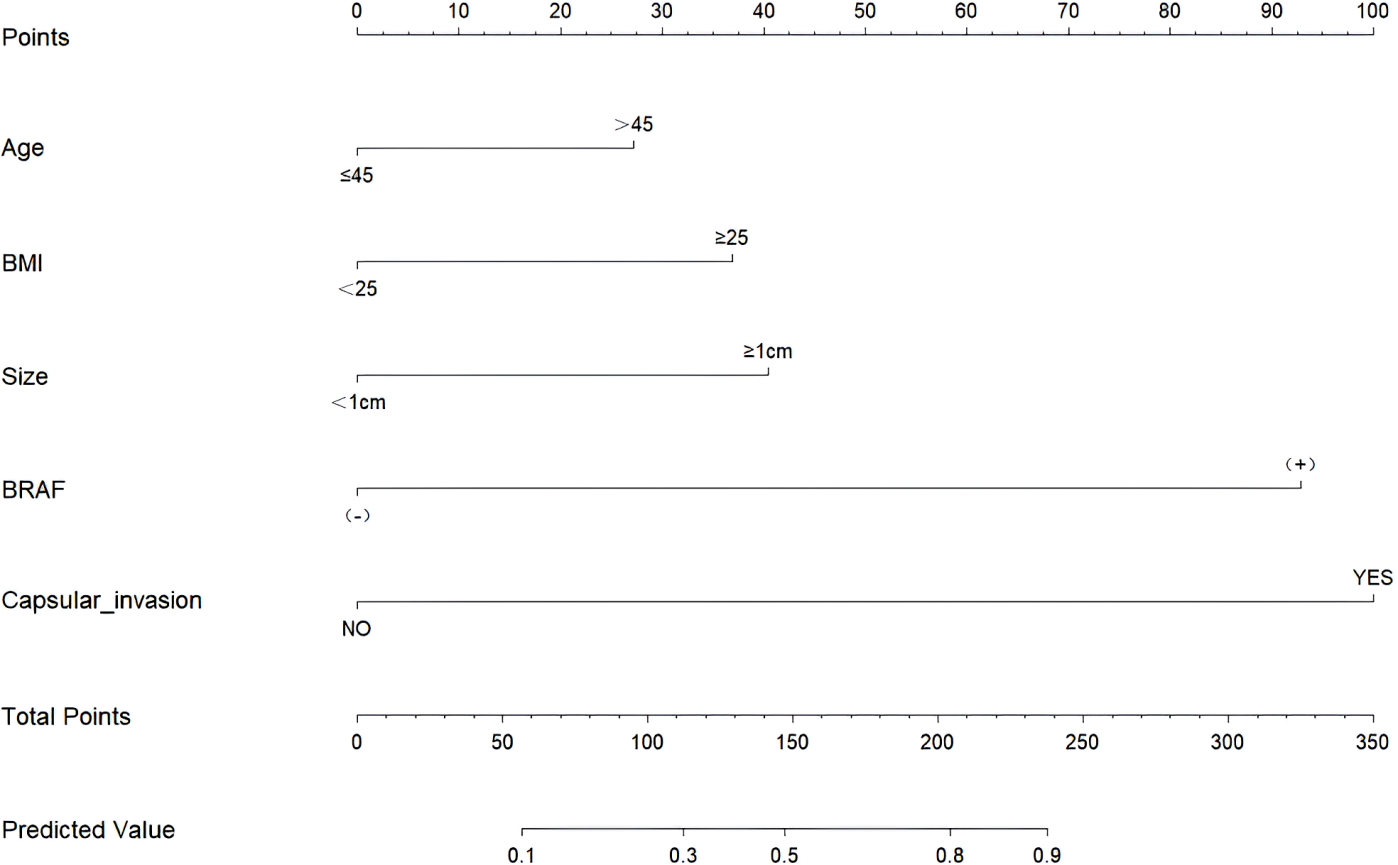
Nomogram for predicting lymph node metastasis in the central region. BMI, Body Mass Index.

**Figure 2:**
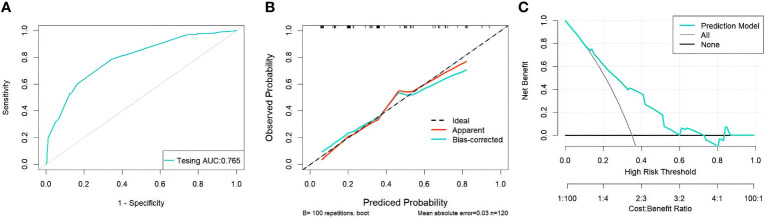
**(A)** ROC curves for validation cohorts **(B)** calibration curves for validation cohorts **(C)** decision curves for validation cohorts.ROC, receiver operating characteristic; AUC, area under the curve.

Through the multivariate logistic regression analysis, the predictive model was illustrated as a nomogram (**Figure 1**). The AUC value of ROC curve for the nomogram in the training and validation cohorts was 0.791 (95% CI: 0.735–0.846) and 0.765 (95% CI: 0.677–0.852), respectively, demonstrating significant discriminatory capacity (**Table 2**). The multivariate model displayed superior predictive (**Table 3**, **Figure 2**). The calibration curve indicated strong agreement between the predicted values and actual observations and the Brier score is 0.020919 (**Figure 2**). The decision curve analysis demonstrated robust clinical usefulness (**Figure 2**).

**Table 2:** AUC (95% CI) for predictors of lymph node metastasis in the central region in the training and validation cohorts.

Characteristics	Validation AUC (95%CI)
Age	0.613 (0.526,0.699)
BMI	0.603 (0.512,0.695)
Size	0.627 (0.547,0.708)
Capsular invasion	0.540 (0.486,0.594)
BRAF	0.560 (0.532,0.668)
Multivariate model	0.765 (0.677,0.852)

BMI, Body Mass Index; 95% CI, 95% confidence interval.

**Table 3:** Prediction performance of nomogram in training and validation cohorts.

Parameters	Training	Testing
ACC	0.912	0.806
Sen	0.765	0.833
Spe	0.952	0.861
PPV	0.813	0.556
NPV	0.937	0.963

AUC, area under the curve; ACC, accuracy; Sen, sensitivity; Spe, specificity; PPV, positive predictive value; NPV, negative predictive value.

### 3.5 Survival outcomes

**Figure 3:**
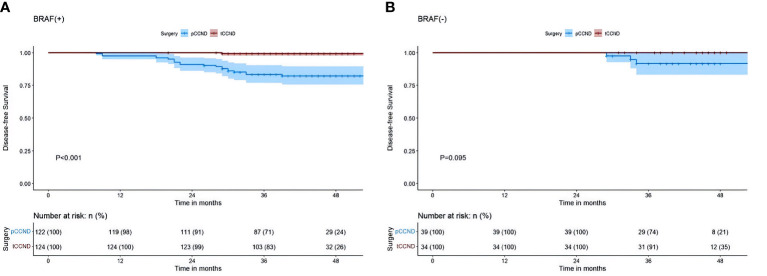
**(A)** Kaplan–Meier survival analysis of DFS between pCCND and tCCND in PTMC patients with *BRAF* V600E mutation **(B)** Kaplan–Meier survival analysis of DFS between pCCND and tCCND in PTMC patients with *BRAF* V600E wild.

**Figure 4:**
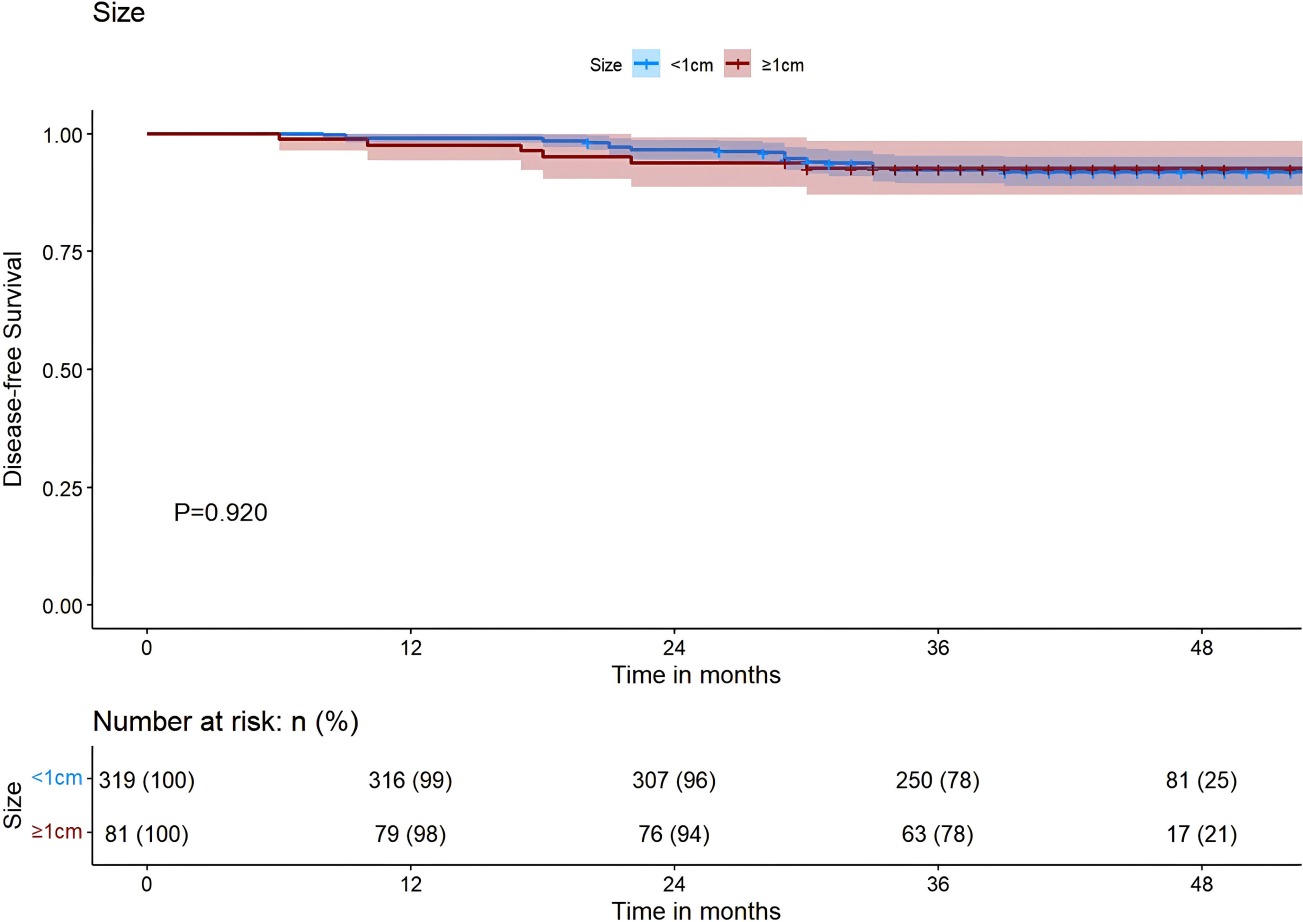
Kaplan–Meier survival analysis of DFS between sizes ≥1 and <1 cm. DFS, Disease-free-survival.

The results of our study revealed that, in PTMC patients with the *BRAF* V600E mutation, those treated with tCCND achieved a significantly improved 3-year DFS compared with those who underwent pCCND (3-year DFS was 83.1% vs. 71.3%, P<0.001) (**Figure 3A**). However, for patients harboring the *BRAF* V600E wild-type allele, the 3-year DFS for those treated with tCCND didn’t display significant improvement when compared with those treated with pCCND (3-year DFS was 91.2% vs. 74.4%, P=0.095) (**Figure 3B**). An analysis of patient survival with different tumor sizes was conducted and revealed no statistically significant difference in 3-year DFS between patients with tumor sizes ≥1 and <1 cm (the 3-year DFS was 77.8% vs. 78.4%, P=0.920) (**Figure 4**).

## 4 Discussion

In 2020, the global incidence of thyroid cancer was documented to reach 586,000 cases, ranking 9th among all cancers. For women, the global incidence of thyroid cancer is 10.1 per 100,000 individuals, thrice higher than that of men. Therefore, examining the risk factors of CNLM in patients with PTC and guiding surgical interventions are essential.

The incidence and mortality rates of thyroid cancer are higher in women than men. We observed a predominance of females over males among the patients we included, with a ratio of approximately 3.4:1. Through univariate analysis, we did not find a correlation between gender and CNLM (P=0.478).Earlier studies have shown that estrogen is implicated in the pathogenesis of female thyroid cancer. Estrogen is an effective growth factor for thyroid cells, promoting growth via membrane-bound receptors. These receptors are connected with the tyrosine kinase signaling pathways, namely MAPK and PI3K. In PTC, these pathways might be activated through chromosomal rearrangement of the tyrosine receptor kinase TRKA by RET/PTC genes or *BRAF* mutations. For female patients, elevated estrogen levels can stimulate relevant signaling transduction pathways to become more significant. Furthermore, estrogen is vital in angiogenesis and metastasis, directly influencing the prognosis of thyroid cancer (13). Similarly, a relevant epidemiological study has identified a significant correlation between thyroid cancer risk and hormonal factors. Estrogen contributes to the etiology of thyroid cancer; however, these correlations have not reached consensus (14).

Age is an independent risk factor for lymph node metastasis in patients with PTC and a primary prognostic factor for the risk of recurrence (15). In previous relevant studies, age was categorized at 45 or 55 years to assess its impact on the development of lymph node metastasis in patients with PTC, leading to divergent conclusions. As for the age that dichotomized by 45 years, many past meta-analyses indicated that younger patients with PTC exhibited a higher rate of developing CLNM. Hence, for younger patients, considering central lymph node dissections is critical (16). However, upon further subdivision of age, patients <20 and >60 years displayed higher lymph node recurrence rates. Notably, patients aged >60 years, regardless of the clinical pathological characteristics of PTC during their initial surgery, demonstrated poor prognoses (17). In our study, using 45 years as the threshold for age division, univariate analysis revealed that exceeding the threshold was associated with CNLM (P=0.002). Additionally, multivariate analysis identified a significant correlation between age >45 years and CNLM (OR=1.645). Therefore, when considering age as an independent risk factor, we believe that analyzing with larger sample sizes and more refined age groups is critical for reliable conclusions.

The global obesity rate has reached approximately 40%, profoundly impacting people’s physical and mental well-being (18). Obesity is an independent risk factor associated with various cancers, and substantial evidence suggests its correlation with that of the thyroid (19). For patients with PTC, obesity might significantly contribute to the rapid increase in the incidence of PTC. Furthermore, obesity increases the rate of CLNM and elevates the number and size of positive lymph nodes (20). The American Thyroid Association guidelines previously recognized the number of lymph node metastases and the maximum diameter of positive lymph nodes as critical criteria for evaluating the recurrence risk in PTC. Patients with PTC having beyond five lymph node metastases had a higher recurrence rate than those with fewer than five. Furthermore, those with positive lymph node metastases exceeding 3 cm have a higher recurrence rate (8). Our results align with those of previous studies, indicating that obese patients have an increased risk of CLNM compared with their non-obese counterparts (OR=2.006). Obese patients tend to have a shorter neck, complicating lymph node dissection during surgical interventions and posing a risk of insufficient lymph node dissection. Thus, surgeons should be particularly cautious when dissecting central lymph nodes in obese patients.

The impact of HT on patients with PTC remains highly controversial. Earlier studies have revealed that PTC combined with HT is frequently observed in female patients, and the thyroid-stimulating hormone level in the HT group surpasses that in patients with PTC alone. HT is not a related risk factor for CLNM in patients with PTC (21). However, studies have shown that patients with concurrent PTC and HT tend toward a more favorable prognosis, and autoimmune thyroiditis protects against thyroid cancer (22). Our univariate analysis revealed no correlation between HT in patients with PTC and CLNM (P=0.890<0.05).

Thyroid capsular invasion denotes a condition where the tumor adheres to the junction of the thyroid and adjacent soft tissues, invading beyond the thyroid into surrounding fibers, adipose tissue, or skeletal muscle tissue (23). The invasion of the thyroid capsule by a tumor is among the most prevalent and recognized risk factors for CLNM and lateral lymph node metastasis in patients with thyroid cancer. Similarly, we concluded that capsular invasion increases the risk of CLNM in patients with PTC.

Tumor multifocality was once considered a risk factor for CNLM in patients with PTC. Multifocality has been associated with lymph node metastasis in patients with PTC (24). Furthermore, the number of tumor lesions is more significant than their location. An increase in the number of tumors elevates the risk of CNLM in patients with PTC (25). In contrast, our study revealed no significant relationships between the number of lesions and CNLM (P=0.355>0.05). We believe that this discrepancy may arise from the limited sample size in our study.

The *BRAF* gene on human chromosome 7 is a primary subtype of the RAF kinase family and is involved in the RAS-RAF-MEK-ERK/MAPK signal transduction pathway. It triggers tumorigenesis by activating the MAPK pathway. Persistent activation of the RAF/MEK/ERK cascade in the MAPK pathway by the *BRAF* V600E mutation promotes cell proliferation and inhibits differentiation and apoptosis. This mutation arises from a T1799A point mutation in its exon, causing the substitution of valine (V) with glutamic acid (E). Such a mutation causes cells to lose their ability to undergo normal apoptosis and triggers tumorigenesis (26). Similarly, the *BRAF* V600E mutation may decrease the expression of immune/inflammatory response genes in patients with PTC, indicating further roles of an immune escape mechanism in its pathogenesis and invasiveness. The rate of the *BRAF* V600E mutation in patients with PTC ranges from 25–82.3%, whereas it is absent in other types of thyroid tumors (27). Preoperative FNA and *BRAF* gene testing benefit PTC diagnosis, and they are extensively used for risk stratification in patients with ultrasonographically suspicious malignant thyroid nodules. Previous studies indicate that the *BRAF* V600E mutation can be an independent predictor for CNLM in patients with PTC. Moreover, a non-mutated *BRAF* V600E may be used to predict the absence of CLNM (28).

In our analysis of the 400 patients with PTC who underwent preoperative FNA+*BRAF* V600E testing, we identified a *BRAF* V600E mutation rate of 79% (316 cases). The univariate analysis revealed a significant association between the *BRAF* V600E mutation in these patients and CLNM (P<0.0001<0.05), similar to the multivariate analysis (OR=3.568, P<0.0001<0.05). However, the mechanism through which *BRAF* V600E affects CLNM and its associated prognosis remains unelucidated; therefore, more clinical data and basic medical research are needed.

The size of the primary tumor is involved in assessing clinical and pathological characteristics and in determining prognosis and mortality rates. It is a critical component in TNM staging, with lesions >1 cm having increasing invasiveness as their size increases (29). In our study, patients with PTC whose tumors were ≥1 cm showed a higher rate of CLNM than those with tumors <1 cm (OR=3.731). Therefore, we suggest a more meticulous dissection of central lymph nodes in patients with PTC having tumor sizes >1 cm.

Papillary carcinoma measuring <1 cm is termed microcarcinoma (PTMC). While its mortality and recurrence rates are relatively low, CLNM rates of PTMC range between 24.1% and 64.1% (30). The American Thyroid Association guidelines (2015) indicate that only a lobectomy is needed for patients with no extrathyroidal invasion and with cN0 central lymph nodes. Conversely, for those with cN0 central lymph nodes who are already at T3/T4 stages or with involved lateral cervical lymph nodes, pCCND is recommended. The European Society for Medical Oncology guidelines (2019) recommend pCCND for patients with cN0 DTC with a history of childhood head and neck radiotherapy, a history of familial thyroid cancer, cytological invasive characteristics, multifocal cancer, suspected extrathyroidal invasion, or a tumor diameter exceeding 4 cm. Additionally, the National Comprehensive Cancer Network guidelines (2022) do not recommend pCCND for patients. Guidelines in China suggest that for DTC patients, provided the parathyroid glands and the recurrent laryngeal nerve are effectively preserved during surgery, a minimum of ipsilateral central lymph node dissection should be performed.

In earlier studies, Efstathios T Pavlidis and Theodoros E Pavlidis proposed that patients with cN0 PTMC, when presenting risk factors for recurrence and diffuse lymph node metastasis (extrathyroidal invasion/*BRAF* gene mutation), should undergo pCCND (31). A retrospective analysis involving 243 patients with cN0 PTMC revealed that the *BRAF* V600E mutation is the primary risk factor for CLNM in those with cN0. This mutation was identified as a significant independent risk factor associated with a poor prognosis. Therefore, pCCND is recommended for patients with PTMC having the *BRAF* V600E mutation (32).

A meta-analysis including 37,355 patients across seven countries verified that the preoperative evaluation of *BRAF* V600E mutations through fine-needle aspiration biopsy is conducive to predicting CLNM in cN0 PTC patients. This insight aids surgeons in assessing the lymph node status of patients with cN0 PTC, directing the surgical approaches for central lymph node dissection (33). These studies have established the predictive ability of the *BRAF* V600E mutation for CLNM; however, examining the implications of mutation on disease recurrence, survival, and prognosis is lacking. The conclusions of the above studies align with our observations, as we recommend pCCND for patients with cN0 PTC having the BRAF V600E mutation.

In our study, a 3-year postoperative follow-up was conducted for all patients starting from the date of surgery. During follow-ups, we discovered that patients with PTMC having the *BRAF* V600E mutation who received tCCND displayed a favorable clinical outcome than those who received pCCND. All patients with PTMC in our study underwent central lymph node dissection, resulting in a CLNM rate lower than that of non-PTMC patients (28.2% vs. 61.7%). Similarly, the suboptimal rate was inferior to the previously reported statistics (64%) (34). Despite the reduced CLNM rate, we believe that among patients with PTMC, those with cN1 having the *BRAF* V600E mutation should receive tCCND. Additionally, in patients with PTMC, tCCND tends to cause a lower recurrence rate and a better prognosis.

This study investigated certain factors contributing to CLNM in patients with PTC via univariate analysis and established a relevant predictive model using multivariate analysis of the identified significant factors (P<0.05), which can guide surgeons in selecting the appropriate surgical approaches for patients with PTC and enhance prognosis. However, our research has some limitations. Firstly, the research included only 400 patients with PTC. Secondary, although a standardized surgical protocol was followed, variations in surgical approaches performed by different doctors might disturb the accuracy of data analysis with unavoidable biases included. Third, most patients in this study are from the same continent—Asia. Finally, given that all patients were recruited from a single center with a retrospective nature, future investigations with larger sample size across multiple centers are needed in the future.

## 5 Conclusion

In conclusion, age >45 years, body mass index ≥25, tumor size ≥1 cm, BRAF V600E mutation, and capsular invasion are risk factors for CLNM in patients with PTC. For PTCM patients with BRAF V600E mutation, therapeutic central lymph node dissection offers a better prognosis than prophylactic central lymph node dissection. Therefore, therapeutic central lymph node dissection may be more appropriate than prophylactic central lymph node dissection for PTMC patients with BRAF V600E mutation.

## Data availability statement

The raw data supporting the conclusions of this article will be made available by the authors, without undue reservation.

## Ethics statement

The studies involving humans were approved by Ethics Committee of Provincial Hospital of Shandong First Medical University. The studies were conducted in accordance with the local legislation and institutional requirements. Written informed consent for participation in this study was provided by the participants’ legal guardians/next of kin. Written informed consent was obtained from the individual(s) for the publication of any potentially identifiable images or data included in this article.

## Author contributions

JD: Writing – original draft. QY: Writing – review & editing. YS: Writing – original draft. PS: Writing – original draft. HX: Writing – original draft. XC: Writing – original draft. TD: Writing – original draft. WS: Writing – original draft. YW: Writing – original draft. ZS: Writing – original draft. XS: Writing – review & editing. XT: Writing – review & editing.
