# Supplementary material for: Risk factors for central lymph node metastasis in patients with papillary thyroid carcinoma: a retrospective study
Source: Front Endocrinol (Lausanne). 2023 Nov 17;14:1288527. doi: 10.3389/fendo.2023.1288527 (PMC10690810; doi:10.3389/fendo.2023.1288527)
Supplement: Supplementary file 1 [file Table_1.docx]

Supplement Table 1 Univariate analysis for predicting lymph node metastasis in the central region

| ***Characteristics*** | ***YES (n=98)*** | ***NO (n=182)*** | ***P*** |
| --- | --- | --- | --- |
| Sex |  |  | 0.478 |
| Female | 84(85.71%) | 150(82.42%) |  |
| Male | 14(14.29%) | 32(17.58%) |  |
| Age |  |  | <0.001 |
| ≤45 | 55(56.12%) | 140(76.92%) |  |
| ＞45 | 43(43.88%) | 42(23.08%) |  |
| BMI |  |  | 0.001 |
| ＜25 | 37(37.76%) | 106(58.24%) |  |
| ≥25 | 61(62.24%) | 76(41.76%) |  |
| Hashimoto’s thyroiditis |  |  | 0.606 |
| YES | 32(32.65%) | 54(29.67%) |  |
| NO | 66(67.35%) | 128(70.33%) |  |
| Focality |  |  | 0.469 |
| Multifocal | 27(27.55%) | 43(23.63%) |  |
| Unifocal | 71(72.45%) | 139(76.37%) |  |
| Size |  |  | <0.001 |
| ＜1cm | 63(64.29%) | 159(87.36%) |  |
| ≥1cm | 35(35.71%) | 23(12.64%) |  |
| Capsular invasion |  |  | <0.001 |
| YES | 11(11.22%) | 2(1.10%) |  |
| NO | 87(88.78%) | 180(98.90%) |  |
| BRAF |  |  | <0.001 |
| (+) | 90(91.84%) | 133(73.08%) |  |
| (-) | 8(8.16%) | 49(26.92%) |  |

BMI, Body Mass Index.

Supplement Table 2 Baseline characteristics of the training and validation cohorts

| ***Characteristics*** | ***Training(n=280)*** | ***Validation (n=120)*** | ***P value*** |
| --- | --- | --- | --- |
| Sex |  |  | 0.069 |
| Female | 234(83.57%) | 91(75.83%) |  |
| Male | 46(16.43%) | 29(24.17%) |  |
| Age |  |  | 0.361 |
| ≤45 | 195(69.64%) | 89(74.17%) |  |
| ＞45 | 85(30.36%) | 31(25.83%) |  |
| BMI |  |  | 0.727 |
| ＜25 | 143(51.07%) | 59(49.17%) |  |
| ≥25 | 137(48.93%) | 61(50.83%) |  |
| Hashimoto’s thyroiditis |  |  | 0.325 |
| YES | 86(30.71%) | 31(25.83%) |  |
| NO | 194(69.29%) | 89(74.17%) |  |
| Focality |  |  | 0.299 |
| Multifocal | 70(25.00%) | 36(30.00%) |  |
| Unifocal | 210(75.00%) | 84(70.00%) |  |
| Size |  |  | 0.724 |
| ＜1cm | 222(79.29%) | 97(80.83%) |  |
| ≥1cm | 58(20.71%) | 23(19.17%) |  |
| Central lymph node metastasis |  |  | 1.000 |
| YES | 98(35.00%) | 42(35.00%) |  |
| NO | 182(65.00%) | 78(65.00%) |  |
| Capsular invasion |  |  | 0.406 |
| YES | 13(4.64%) | 8(6.67%) |  |
| NO | 267(95.36%) | 112(93.33%) |  |
| BRAF |  |  | 0.630 |
| (+) | 223(79.64%) | 93(77.50%) |  |
| (-) | 57(20.36%) | 27(22.50%) |  |
| FT3 | 4.16±0.55 | 4.21±0.48 | 0.385 |
| FT4 | 12.22±6.58 | 11.43±1.20 | 0.192 |
| TSH | 2.01±1.41 | 2.40±2.92 | 0.075 |
| CEA | 1.76±5.73 | 1.50±0.99 | 0.629 |

BMI, Body Mass Index; FT3, free triiodothyronine; FT4, free thyroxin; TSH, thyroid stimulating hormone; CEA, carcinoembryonic antigen.

Supplement Table 3 Multivariate analysis for predicting lymph node metastasis in the central region

| ***Characteristic*** | ***Regression coefficient*** | ***OR (95%CI)*** | ***P value*** |
| --- | --- | --- | --- |
| Age(＞45) | 0.767 | 2.153 (1.166, 3.974) | 0.014 |
| BMI(≥25) | 0.773 | 2.165 (1.224,3.829) | 0.008 |
| Size(≥1cm) | 1.139 | 3.123 (1.606,6.074) | 0.001 |
| Capsular invasion(Yes) | 3.374 | 29.183 (4.152,205.138) | 0.001 |
| BRAF(+) | 2.070 | 7.924 (2.683,23.402) | <0.001 |

BMI, Body Mass Index; OR, odds ratio; 95% CI, 95% confidence interval;
